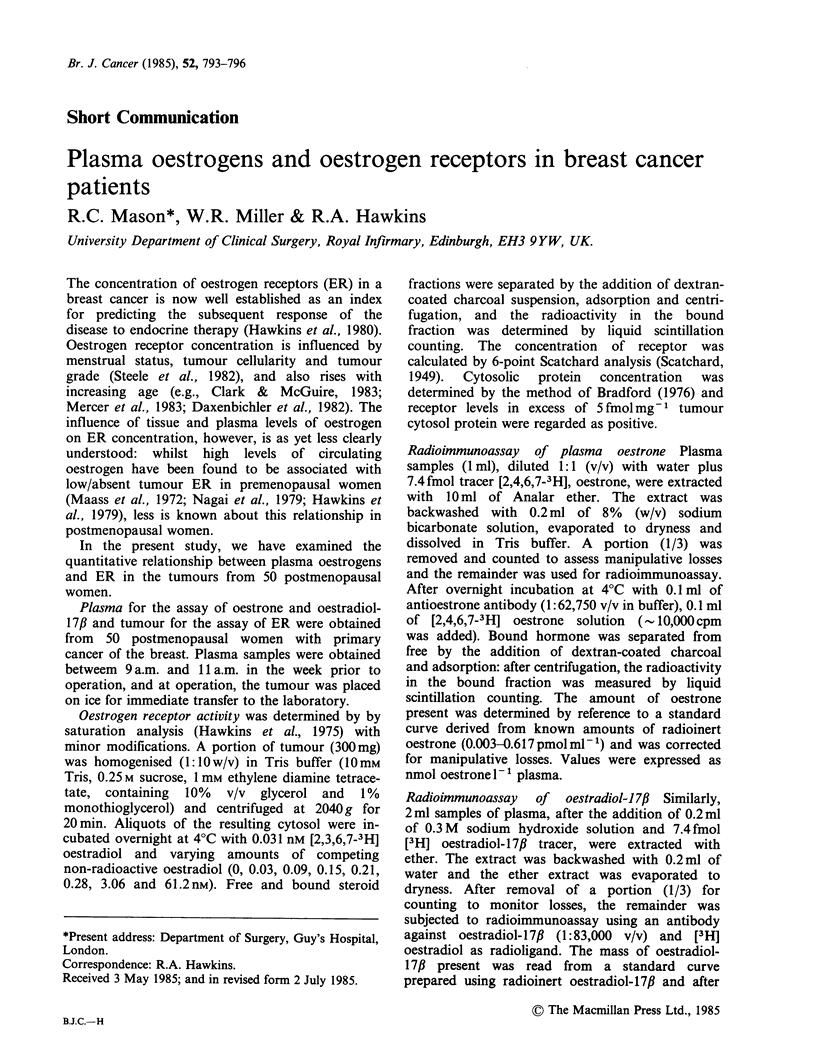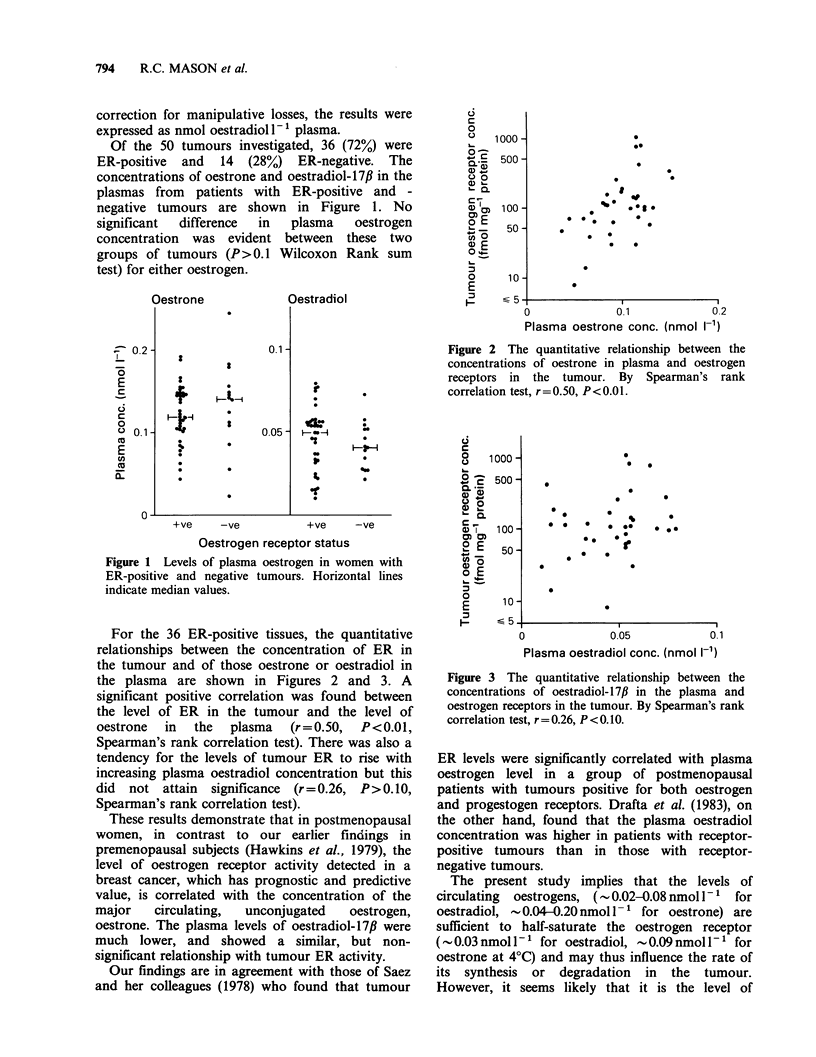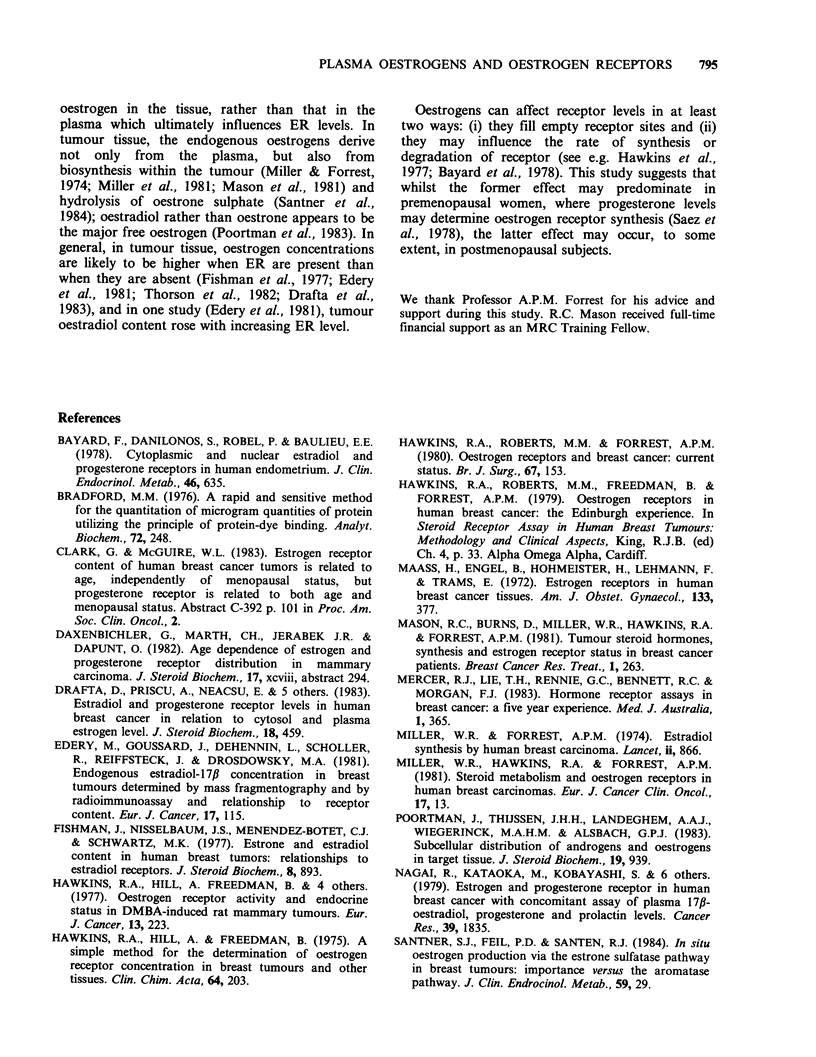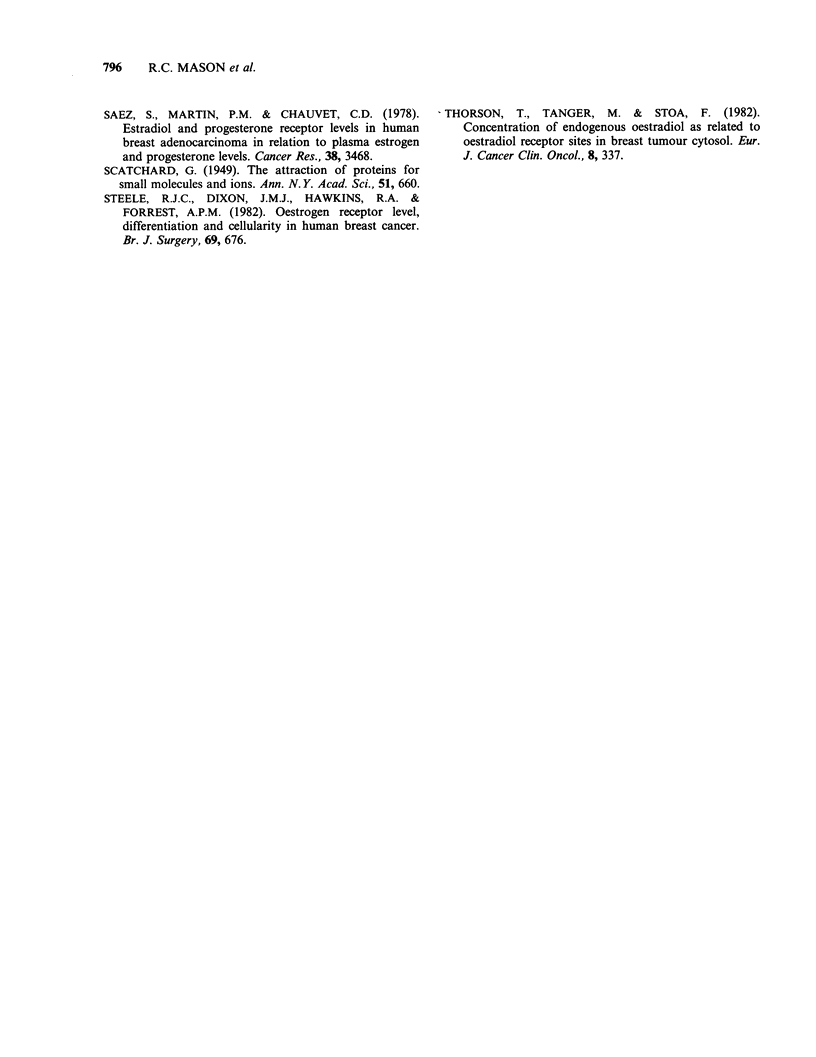# Plasma oestrogens and oestrogen receptors in breast cancer patients.

**DOI:** 10.1038/bjc.1985.260

**Published:** 1985-11

**Authors:** R. C. Mason, W. R. Miller, R. A. Hawkins


*Br. J. Cancer* (1985), **52**, 793–796

### Short Communication

### Plasma oestrogens and oestrogen receptors in breast cancer patients

R.C. Mason*, W.R. Miller & R.A. Hawkins

*University Department of Clinical Surgery, Royal Infirmary, Edinburgh, EH3 9YW, UK.*

The concentration of oestrogen receptors (ER) in a breast cancer is now well established as an index for predicting the subsequent response of the disease to endocrine therapy (Hawkins *et al.*, 1980). Oestrogen receptor concentration is influenced by menstrual status, tumour cellularity and tumour grade (Steele *et al.*, 1982), and also rises with increasing age (e.g., Clark & McGuire, 1983; Mercer *et al.*, 1983; Daxenbichler *et al.*, 1982). The influence of tissue and plasma levels of oestrogen on ER concentration, however, is as yet less clearly understood: whilst high levels of circulating oestrogen have been found to be associated with low/absent tumour ER in premenopausal women (Maass *et al.*, 1972; Nagai *et al.*, 1979; Hawkins *et al.*, 1979), less is known about this relationship in postmenopausal women.

In the present study, we have examined the quantitative relationship between plasma oestrogens and ER in the tumours from 50 postmenopausal women.

*Plasma* for the assay of oestrone and oestradiol-17$\beta$ and tumour for the assay of ER were obtained from 50 postmenopausal women with primary cancer of the breast. Plasma samples were obtained betweem 9 a.m. and 11 a.m. in the week prior to operation, and at operation, the tumour was placed on ice for immediate transfer to the laboratory.

*Oestrogen receptor activity* was determined by by saturation analysis (Hawkins *et al.*, 1975) with minor modifications. A portion of tumour (300 mg) was homogenised (1:10 w/v) in Tris buffer (10 mM Tris, 0.25 M sucrose, 1 mM ethylene diamine tetracetate, containing 10% v/v glycerol and 1% monothioglycerol) and centrifuged at 2040 $g$ for 20 min. Aliquots of the resulting cytosol were incubated overnight at 4°C with 0.031 nM [2,3,6,7-$^3$H] oestradiol and varying amounts of competing non-radioactive oestradiol (0, 0.03, 0.09, 0.15, 0.21, 0.28, 3.06 and 61.2 nM). Free and bound steroid fractions were separated by the addition of dextran-coated charcoal suspension, adsorption and centrifugation, and the radioactivity in the bound fraction was determined by liquid scintillation counting. The concentration of receptor was calculated by 6-point Scatchard analysis (Scatchard, 1949). Cytosolic protein concentration was determined by the method of Bradford (1976) and receptor levels in excess of 5 fmol mg$^{-1}$ tumour cytosol protein were regarded as positive.

*Radioimmunoassay of plasma oestrone* Plasma samples (1 ml), diluted 1:1 (v/v) with water plus 7.4 fmol tracer [2,4,6,7-$^3$H], oestrone, were extracted with 10 ml of Analar ether. The extract was backwashed with 0.2 ml of 8% (w/v) sodium bicarbonate solution, evaporated to dryness and dissolved in Tris buffer. A portion (1/3) was removed and counted to assess manipulative losses and the remainder was used for radioimmunoassay. After overnight incubation at 4°C with 0.1 ml of antioestrone antibody (1:62,750 v/v in buffer), 0.1 ml of [2,4,6,7-$^3$H] oestrone solution (~10,000 cpm was added). Bound hormone was separated from free by the addition of dextran-coated charcoal and adsorption: after centrifugation, the radioactivity in the bound fraction was measured by liquid scintillation counting. The amount of oestrone present was determined by reference to a standard curve derived from known amounts of radioinert oestrone (0.003–0.617 pmol ml$^{-1}$) and was corrected for manipulative losses. Values were expressed as nmol oestrone l$^{-1}$ plasma.

*Radioimmunoassay of oestradiol-17$\beta$* Similarly, 2 ml samples of plasma, after the addition of 0.2 ml of 0.3 M sodium hydroxide solution and 7.4 fmol [$^3$H] oestradiol-17$\beta$ tracer, were extracted with ether. The extract was backwashed with 0.2 ml of water and the ether extract was evaporated to dryness. After removal of a portion (1/3) for counting to monitor losses, the remainder was subjected to radioimmunoassay using an antibody against oestradiol-17$\beta$ (1:83,000 v/v) and [$^3$H] oestradiol as radioligand. The mass of oestradiol-17$\beta$ present was read from a standard curve prepared using radioinert oestradiol-17$\beta$ and after

*Present address: Department of Surgery, Guy's Hospital, London.
Correspondence: R.A. Hawkins.
Received 3 May 1985; and in revised form 2 July 1985.

© The Macmillan Press Ltd., 1985

correction for manipulative losses, the results were expressed as nmol oestradiol $l^{-1}$ plasma.

Of the 50 tumours investigated, 36 (72%) were ER-positive and 14 (28%) ER-negative. The concentrations of oestrone and oestradiol-17$\beta$ in the plasmas from patients with ER-positive and -negative tumours are shown in Figure 1. No significant difference in plasma oestrogen concentration was evident between these two groups of tumours ($P>0.1$ Wilcoxon Rank sum test) for either oestrogen.

**Figure 1** Levels of plasma oestrogen in women with ER-positive and negative tumours. Horizontal lines indicate median values.

For the 36 ER-positive tissues, the quantitative relationships between the concentration of ER in the tumour and of those oestrone or oestradiol in the plasma are shown in Figures 2 and 3. A significant positive correlation was found between the level of ER in the tumour and the level of oestrone in the plasma ($r=0.50$, $P<0.01$, Spearman's rank correlation test). There was also a tendency for the levels of tumour ER to rise with increasing plasma oestradiol concentration but this did not attain significance ($r=0.26$, $P>0.10$, Spearman's rank correlation test).

These results demonstrate that in postmenopausal women, in contrast to our earlier findings in premenopausal subjects (Hawkins et al., 1979), the level of oestrogen receptor activity detected in a breast cancer, which has prognostic and predictive value, is correlated with the concentration of the major circulating, unconjugated oestrogen, oestrone. The plasma levels of oestradiol-17$\beta$ were much lower, and showed a similar, but non-significant relationship with tumour ER activity.

Our findings are in agreement with those of Saez and her colleagues (1978) who found that tumour

**Figure 2** The quantitative relationship between the concentrations of oestrone in plasma and oestrogen receptors in the tumour. By Spearman's rank correlation test, $r=0.50$, $P<0.01$.

**Figure 3** The quantitative relationship between the concentrations of oestradiol-17$\beta$ in the plasma and oestrogen receptors in the tumour. By Spearman's rank correlation test, $r=0.26$, $P<0.10$.

ER levels were significantly correlated with plasma oestrogen level in a group of postmenopausal patients with tumours positive for both oestrogen and progestogen receptors. Drafta et al. (1983), on the other hand, found that the plasma oestradiol concentration was higher in patients with receptor-positive tumours than in those with receptor-negative tumours.

The present study implies that the levels of circulating oestrogens, ($\sim$0.02–0.08 nmol $l^{-1}$ for oestradiol, $\sim$0.04–0.20 nmol $l^{-1}$ for oestrone) are sufficient to half-saturate the oestrogen receptor ($\sim$0.03 nmol $l^{-1}$ for oestradiol, $\sim$0.09 nmol $l^{-1}$ for oestrone at 4°C) and may thus influence the rate of its synthesis or degradation in the tumour. However, it seems likely that it is the level of oestrogen in the tissue, rather than that in the plasma which ultimately influences ER levels. In tumour tissue, the endogenous oestrogens derive not only from the plasma, but also from biosynthesis within the tumour (Miller & Forrest, 1974; Miller *et al.*, 1981; Mason *et al.*, 1981) and hydrolysis of oestrone sulphate (Santner *et al.*, 1984); oestradiol rather than oestrone appears to be the major free oestrogen (Poortman *et al.*, 1983). In general, in tumour tissue, oestrogen concentrations are likely to be higher when ER are present than when they are absent (Fishman *et al.*, 1977; Edery *et al.*, 1981; Thorson *et al.*, 1982; Drafta *et al.*, 1983), and in one study (Edery *et al.*, 1981), tumour oestradiol content rose with increasing ER level.

Oestrogens can affect receptor levels in at least two ways: (i) they fill empty receptor sites and (ii) they may influence the rate of synthesis or degradation of receptor (see e.g. Hawkins *et al.*, 1977; Bayard *et al.*, 1978). This study suggests that whilst the former effect may predominate in premenopausal women, where progesterone levels may determine oestrogen receptor synthesis (Saez *et al.*, 1978), the latter effect may occur, to some extent, in postmenopausal subjects.

We thank Professor A.P.M. Forrest for his advice and support during this study. R.C. Mason received full-time financial support as an MRC Training Fellow.

### References

BAYARD, F., DANILONOS, S., ROBEL, P. & BAULIEU, E.E. (1978). Cytoplasmic and nuclear estradiol and progesterone receptors in human endometrium. *J. Clin. Endocrinol. Metab.*, **46**, 635.

BRADFORD, M.M. (1976). A rapid and sensitive method for the quantitation of microgram quantities of protein utilizing the principle of protein-dye binding. *Analyt. Biochem.*, **72**, 248.

CLARK, G. & McGUIRE, W.L. (1983). Estrogen receptor content of human breast cancer tumors is related to age, independently of menopausal status, but progesterone receptor is related to both age and menopausal status. Abstract C-392 p. 101 in *Proc. Am. Soc. Clin. Oncol.*, **2**.

DAXENBICHLER, G., MARTH, CH., JERABEK J.R. & DAPUNT, O. (1982). Age dependence of estrogen and progesterone receptor distribution in mammary carcinoma. *J. Steroid Biochem.*, **17**, xcviii, abstract 294.

DRAFTA, D., PRISCU, A., NEACSU, E. & 5 others. (1983). Estradiol and progesterone receptor levels in human breast cancer in relation to cytosol and plasma estrogen level. *J. Steroid Biochem.*, **18**, 459.

EDERY, M., GOUSSARD, J., DEHENNIN, L., SCHOLLER, R., REIFFSTECK, J. & DROSDOWSKY, M.A. (1981). Endogenous estradiol-17β concentration in breast tumours determined by mass fragmentography and by radioimmunoassay and relationship to receptor content. *Eur. J. Cancer*, **17**, 115.

FISHMAN, J., NISSELBAUM, J.S., MENENDEZ-BOTET, C.J. & SCHWARTZ, M.K. (1977). Estrone and estradiol content in human breast tumors: relationships to estradiol receptors. *J. Steroid Biochem.*, **8**, 893.

HAWKINS, R.A., HILL, A. FREEDMAN, B. & 4 others. (1977). Oestrogen receptor activity and endocrine status in DMBA-induced rat mammary tumours. *Eur. J. Cancer*, **13**, 223.

HAWKINS, R.A., HILL, A. & FREEDMAN, B. (1975). A simple method for the determination of oestrogen receptor concentration in breast tumours and other tissues. *Clin. Chim. Acta*, **64**, 203.

HAWKINS, R.A., ROBERTS, M.M. & FORREST, A.P.M. (1980). Oestrogen receptors and breast cancer: current status. *Br. J. Surg.*, **67**, 153.

HAWKINS, R.A., ROBERTS, M.M., FREEDMAN, B. & FORREST, A.P.M. (1979). Oestrogen receptors in human breast cancer: the Edinburgh experience. In *Steroid Receptor Assay in Human Breast Tumours: Methodology and Clinical Aspects*, King, R.J.B. (ed) Ch. 4, p. 33. Alpha Omega Alpha, Cardiff.

MAASS, H., ENGEL, B., HOHMEISTER, H., LEHMANN, F. & TRAMS, E. (1972). Estrogen receptors in human breast cancer tissues. *Am. J. Obstet. Gynaecol.*, **133**, 377.

MASON, R.C., BURNS, D., MILLER, W.R., HAWKINS, R.A. & FORREST, A.P.M. (1981). Tumour steroid hormones, synthesis and estrogen receptor status in breast cancer patients. *Breast Cancer Res. Treat.*, **1**, 263.

MERCER, R.J., LIE, T.H., RENNIE, G.C., BENNETT, R.C. & MORGAN, F.J. (1983). Hormone receptor assays in breast cancer: a five year experience. *Med. J. Australia*, **1**, 365.

MILLER, W.R. & FORREST, A.P.M. (1974). Estradiol synthesis by human breast carcinoma. *Lancet*, **ii**, 866.

MILLER, W.R., HAWKINS, R.A. & FORREST, A.P.M. (1981). Steroid metabolism and oestrogen receptors in human breast carcinomas. *Eur. J. Cancer Clin. Oncol.*, **17**, 13.

POORTMAN, J., THIJSSEN, J.H.H., LANDEGHEM, A.A.J., WIEGERINCK, M.A.H.M. & ALSBACH, G.P.J. (1983). Subcellular distribution of androgens and oestrogens in target tissue. *J. Steroid Biochem.*, **19**, 939.

NAGAI, R., KATAOKA, M., KOBAYASHI, S. & 6 others. (1979). Estrogen and progesterone receptor in human breast cancer with concomitant assay of plasma 17β-oestradiol, progesterone and prolactin levels. *Cancer Res.*, **39**, 1835.

SANTNER, S.J., FEIL, P.D. & SANTEN, R.J. (1984). *In situ* oestrogen production via the estrone sulfatase pathway in breast tumours: importance *versus* the aromatase pathway. *J. Clin. Endrocinol. Metab.*, **59**, 29.

SAEZ, S., MARTIN, P.M. & CHAUVET, C.D. (1978). Estradiol and progesterone receptor levels in human breast adenocarcinoma in relation to plasma estrogen and progesterone levels. *Cancer Res.*, **38**, 3468.

SCATCHARD, G. (1949). The attraction of proteins for small molecules and ions. *Ann. N.Y. Acad. Sci.*, **51**, 660.

STEELE, R.J.C., DIXON, J.M.J., HAWKINS, R.A. & FORREST, A.P.M. (1982). Oestrogen receptor level, differentiation and cellularity in human breast cancer. *Br. J. Surgery*, **69**, 676.

THORSON, T., TANGER, M. & STOA, F. (1982). Concentration of endogenous oestradiol as related to oestradiol receptor sites in breast tumour cytosol. *Eur. J. Cancer Clin. Oncol.*, **8**, 337.